# A Retrospective Study on Risk Factors for Urinary Tract Infection in Patients with Intracranial Cerebral Hemorrhage

**DOI:** 10.1155/2020/1396705

**Published:** 2020-01-28

**Authors:** Jingsong Mu, Chaomin Ni, Ming Wu, Wenxiang Fan, Zheng Liu, Fengjuan Xu, Lei Liu

**Affiliations:** Department of Rehabilitation Medicine, The First Affiliated Hospital of USTC, Division of Life Sciences and Medicine, University of Science and Technology of China, Hefei, Anhui 230001, China

## Abstract

**Objective:**

This study aimed to explore the risk factors of urinary tract infection (UTI) in patients with intracranial cerebral hemorrhage (ICH).

**Design:**

This is a retrospective study, and a total of 77 patients with ICH consecutively admitted to the First Affiliated Hospital of USTC (Anhui Provincial Hospital, Hefei, China) during the period of August 2015 to August 2017 were included. The patients were divided into an UTI group (24 cases) and a non-UTI group (53 cases); patients with UTI were diagnosed according to clinical manifestations, recent urinary routines, and urine culture results. The following information in these two groups was recorded: age, sex, course of disease, side of paralysis, location and type of cerebral hemorrhage, disturbance of consciousness or not, the Brunnstrom stage of paralysed lower limbs, number of basic diseases, whether there were complications (tracheotomy, retention catheterization, pulmonary infection, pressure sore, deep venous thrombosis, etc.), whether rehabilitation interventions were conducted, blood routine, biochemistry index, DIC complete set, urine routine, and urine culture data. Univariate analysis and multivariate logistic regression analysis were used to examine the risk factors of UTI in patients with ICH.

**Results:**

Univariate analysis showed that age, side of paralysis, disturbance of consciousness, the Brunnstrom stage of lower limbs, tracheotomies, retention catheterization, pulmonary infection, leukocyte count, neutrophil proportion, sodium, uric acid, D-dimer, and fibrinogen may be related to UTI in patients with ICH (*P* < 0.05). Regression analysis showed that age (OR (95% CI) = 1.207 (1.022–1.424), *P* < 0.05). Regression analysis showed that age (OR (95% CI) = 1.207 (1.022–1.424), *P* < 0.05). Regression analysis showed that age (OR (95% CI) = 1.207 (1.022–1.424), *P* < 0.05). Regression analysis showed that age (OR (95% CI) = 1.207 (1.022–1.424),

**Conclusions:**

Increased age and high D-dimer are independent risk factors for UTI in patients with ICH, while right-sided paralysis is a protective factor for UTI in patients with ICH.

## 1. Introduction

Urinary tract infection (UTI) is not only a significant complication but also a potential risk factor for poor prognosis of stroke. It has been reported that the incidence of UTI in stroke patients is 14.2–34.7% [1–3]. Intracranial cerebral hemorrhage (ICH) is the most severe subtype of stroke [4,5], with a mortality rate of 29% within one year of onset [6], and UTI-induced death accounts for about 20% of total ICH deaths [7]. The incidence of UTI after ICH is 15.1–26.1% [8–10]. The occurrence of UTI not only prolongs hospitalization time and increases the costs of ICH, but also leads to worsening of the disease, which is a heavy economic and psychological burden to families and society [8,11–13]. Therefore, a better understanding of the risk factors of UTI in ICH patients may lead to more timely and effective prevention and intervention measures, which can prevent disease progression and comprehensively improve patient survival and quality of life.

Previous studies have reported that increased age is a risk factor for UTI in ICH patients, and patients over 76 years old account for 28.6% of hospital-acquired UTI, while ICH accounts for 34.2% [11]. However, risk factors of UTI in patients with ICH are largely unclear. Here, a retrospective study was conducted to identify the possible risk factors of UTI in ICH patients. Our results indicate that, in addition to increased age, high D-dimer is an independent risk factor for UTI in patients with ICH, while right-sided paralysis is a protective factor for UTI in patients with ICH.

## 2. Subjects and Methods

### 2.1. Patient Selection

  Inclusion criteria were as follows: (1) diagnosed with cerebral hemorrhage (including subarachnoid hemorrhage) by CT in accordance with the fourth national diagnostic criteria for cerebrovascular disease of 1995 in China [14]; (2) age ≥ 50 years; (3) 1 week ≤ course ≤ 12 weeks; (4) agreed to undergo a blood routine, biochemical analysis, urine routine, urine culture, and other examinations after admission; (5) signed the rehabilitation informed consent form. This study was approved by the Ethics Committee of the First Affiliated Hospital of USTC (Anhui Provincial Hospital).
  Exclusion criteria: (1) clinical data are incomplete; (2) UTI presented before the ICH.

A retrospective analysis was conducted for the study. A total of 77 ICH patients who met the above criteria and admitted to the Department of Rehabilitation Medicine of the First Affiliated Hospital of USTC (Anhui Provincial Hospital) from August 2015 to August 2017 were included in the study. Among them, 47 males and 30 females, ranging in age from 50 to 87 years, were included.

### 2.2. Methods

Information recorded included age, sex, course of disease, paralysis side (left, right, or double), location and type of bleeding (for convenience, we establish the following settings: left ICH: A type; right ICH: B type; ICH involved in the brainstem and ventricle: C type; subarachnoid hemorrhage (SAH): D type), number of underlying diseases, disturbance of consciousness or not, and the motor function of paralysed lower limbs. Brunnstrom staging was used to make assessments: Brunnstrom Stage 1 was set for paralysis of lower limbs with a disturbance of consciousness, Brunnstrom Stage 2 was set for spasm without involuntary activity, and Brunnstrom Stage 3 was set for spasm and involuntary activity. If the patient had bilateral paralysis, Brunnstrom staging of the serious side was considered for the study. Other information recorded at admission included complications (whether a tracheotomy had occurred, retention catheterization, pulmonary infection, pressure sores, thrombosis in lower limb deep veins, etc.), whether rehabilitation intervention was conducted or not after the onset of ICH, blood routine, biochemistry index, DIC complete set, urine routine, and urine culture clinical data.

The patients were divided into a UTI group (24 cases) and a non-UTI group (53 cases). The general data of the two groups are listed in Table 1. The patients of the UTI group had clinical symptoms of UTI at admission, which include urinary tract irritation symptoms, discomfort in perineal and bladder areas, cloudy urine, and even hematuria. The disease was further confirmed by routine urine and urine culture examination [15].

### 2.3. Statistical Analysis

SPSS 21.0 statistical software was used for analysis in this study. An independent sample *t* test was used when the two groups of measurement data were in accordance with normal distribution, and a rank-sum test (Mann–Whitney U test) was used when there was no such accordance. The results are expressed as Mean ± SD. A comparison of the categorical data between the UTI group and the non-UTI group was performed by the chi-square test or Fisher's exact test, and the results are expressed by the number of cases. When analyzing the risk factors of UTI in ICH patients, UTI was used as a dependent variable, and meaningful indicators were used as independent variables after univariate analysis. A multivariate logistic regression analysis was carried out.  *P* < 0.05 was considered statistically significant.

## 3. Results

### 3.1. Comparison of Complications, Secondary Disorders, and Rehabilitation Interventions between the UTI Group and the Non-UTI Group

By analyzing data using univariate analysis, it can be found that there were significant differences between the UTI group and the non-UTI group with respect to consciousness disorders, tracheotomies, retention catheterization, and pulmonary infection (*P* < 0.01), as shown in Table 2, which suggests that these factors may be related to UTI after ICH in patients. Additionally, the Brunnstrom stage of the lower limbs in the UTI group was significantly lower than that in the non-UTI group (*P* < 0.05), suggesting that the Brunnstrom stage of lower limbs may also be related to UTI. However, there was no significant difference in pressure sore, venous thrombosis, and rehabilitation intervention between the UTI group and the non-UTI group (*P* > 0.05), indicating that these factors may not be associated with UTI in patients with ICH.

### 3.2. Comparison of Blood Indices between the UTI Group and the Non-UTI Group

Because blood indices play a key role in the reflection of infection, we compared several blood indices in the UTI and non-UTI groups. As can be inferred from the results using univariate analysis in Table 3, the leukocyte count, neutrophil proportion, D-dimer level, and fibrinogen level in the UTI group were significantly higher than those in the non-UTI group (*P* < 0.01). The levels of serum uric acid and sodium in the UTI group were significantly lower than those in the non-UTI group (*P* < 0.01), while there were no statistically significant differences between the two groups in serum albumin, globulin, blood sugar, creatinine, potassium, calcium, and magnesium (*P* > 0.05). These results suggest that leukocyte count, neutrophil proportion, D-dimer level, and fibrinogen level may be risk factors for UTI in patients with ICH.

### 3.3. Multivariate Analysis of Urinary Tract Infection in Patients with Cerebral Hemorrhage

To further find potential independent risk factors for UTI in patients with ICH, 13 significant variables identified by univariate analysis were introduced into the logistic regression equation. The multivariate analysis results showed that age (OR (95% CI) = 1.207 (1.022–1.424), *P*=0.026), right-sided paralysis (OR (95% CI) = 0.20 (0.001–0.650), *P*=0.028), and D-dimer (OR (95% CI) = 1.403 (1.003–1.961), *P*=0.048) were related to ICH concurrent with UTI, as shown in Table 4.

Our results showed that right-sided paralysis is a protective factor of UTI. To address this question, we reanalyzed the data by taking sex into consideration and compared the infection rate of the left and right urethra in a subgroup analysis. As shown in Tables 5 and 6, there was a significant difference in the incidence of UTI in the different paralysed sides of male patients (*χ*^2^ = 6.873, *P*=0.032), Among them, the incidence of UTI with right paralysis is significantly lower than left, while the incidence of UTI in female with left and right paralysis was not significantly different (*χ*^2^ = 0.772, *P*=0.680).

## 4. Discussion

UTI not only led to reduced exercise during rehabilitation, but also prolonged hospitalization time and reduced the effect of rehabilitation, which is a heavy economic and psychological burden to patients, their families, and society. Therefore, understanding the risk factors of UTI in ICH patients may lead to more effective prevention and intervention with respect to ICH patients. Here, we demonstrated that age, right-sided paralysis, and D-dimer are risk factors associated with UTI in ICH patients and that right-sided paralysis is a protective factor.

Univariate and multivariate analysis showed that increased age was associated with UTI in this study, which is consistent with previous findings suggesting that age (>75 years) is a factor associated with UTI in stroke patients [2,16,17]. With the increase of age, the anatomical barrier and physiological function of the urinary system gradually decline. Increased metabolic disorders, dysregulated immune functions, an imbalance of urine pH and sugar content, and an increase of pathogen adhesion to the bladder wall in aged patients may result in an increased risk of UTI in elderly patients. Therefore, the impact of increased age on UTI after ICH requires attention.

Our results indicate that the location and type of bleeding have no influence on the UTI in patients after ICH, but whether there is any difference in the amount of bleeding between UTI and non-UTI groups is unclear at the current stage. Future studies are required for determining whether the size of hemorrhage is related to UTI in patients after ICH. Since the bleeding data for many patients in our hospital are lacking, it is impossible to analyze the relationship between the size of hemorrhage and the UTI in patients after ICH. We hope that neurologists can record more patient information, including the amount of ICH, so as to provide richer information for later clinical research.

The association of the side of paralysis with respect to UTI in patients with ICH is not clear. Our results showed that right-sided paralysis was related to UTI after ICH. Compared with left-sided paralysis, right-sided paralysis was a protective factor of UTI, and the risk of right-sided paralysis UTI was only 0.020 times that of left-sided paralysis (OR = 0.020), which might be due to hemiplegia slowing down the ipsilateral ureteral peristalsis. The length of the left and right ureter is different in humans. This study found that the infection rate of right paralysis was lower than left, but we neglected the anatomical differences between the males and females. To solve this problem, we conducted a recalculation and subgroup analysis of the infection rate of left and right paralysis by the sex group. Finally, we found that the infection rate of right paralysis in males was lower than left. However, there was no statistically significant difference in the infection rate of the left and right in females, indicating that the right paralysis may be a protective factor for UTI in males. Future studies are required to address this question by increasing the number of patients. An alternative explanation could relate to the shorter anatomical characteristics of the right ureter (shorter than the left ureter). Pathogens such as bacteria and fungi of retrograde urine are easier to remove when right ureter peristalses occur. The left ureter is relatively long, and the slow peristalsis of the left ureter has a greater impact on it. The pathogen stays in the interior for a long time after the occurrence of retrograde urine, allowing it to more easily adhere and reproduce. Interestingly, by dividing ICH patients according to hemorrhage side, brainstem and ventricle, and subarachnoid hemorrhage groups, there were no significant difference in terms of hemorrhage side or location with respect to UTI, which further indicates that the paralysis side may only come from the paralysis itself but not from the hemorrhage side or the location in the brain.

Moreover, our results indicated that D-dimer increased significantly in UTI patients, and D-dimer increased the risk of UTI by 1.403 times (OR = 1.403). D-dimer is a soluble degradation product of fibrin under the action of the fibrinolytic system. The increased concentration of D-dimer indicates that the fibrinolytic activity of fibrin is enhanced in vivo, which could be a factor predicting the occurrence of deep venous thrombosis [18]. However, this activity also increases significantly during bacterial infection and sepsis and is considered an inflammatory marker of UTI [19]. The increase of D-dimer in UTI patients may be because inflammation and coagulation can promote each other. On the one hand, inflammation activates coagulation factors, which leads to the downregulation of the physiological anticoagulation pathway. On the other hand, monocytes are stimulated by preinflammatory cytokines to express tissue factors, which leads to the activation of systemic coagulation [20]. Therefore, the detection of the D-dimer level in clinical work is not only helpful for determining the risk of thrombosis but also helpful in understanding a UTI situation. The appropriate use of anticoagulant and antifibrinolytic drugs according to specific conditions can prevent and block the activation of the coagulation pathway in the early stage, which may help to reduce or delay the severity of UTI. Therefore, the findings that D-dimer increased the risk of UTI may provide novel insight into the use of anticoagulant and antifibrinolytic drugs for treating ICH patients, although further evidence is needed for future studies.

A tracheotomy can improve ventilation and strengthen airway management in ICH patients but can easily cause laryngeal defensive reflex weakening and dysphagia, aspiration of food, and oropharyngeal esophageal secretions, which may result in pulmonary infection [21]. Our study showed that tracheotomies and pulmonary infections were associated with UTI, which is consistent with previous findings [22], and the reasons may relate to a common inflammation mechanism. In addition, our results also showed that disorders of consciousness and the Brunnstrom stage of lower limbs were associated with UTI by univariate analysis, probably because such patients were more likely to stay in bed, which increased the chance of urinary retention and UTI. Furthermore, it was also found using univariate analysis that the levels of serum uric acid and sodium in the UTI group were significantly lower than those in the non-UTI group. This may be because most UTI patients stay longer in bed, which could lead to an increase in renal blood flow as well as an increase in uric acid and sodium excreted through urine [23]. Finally, because of the difference in the anatomical structure of the urethra between males and females, the risk of UTI in females was significantly higher than that in males, as shown previously [9,24]. There was a trend that females were more susceptible than males (13/30 : 11/47), but our results did not demonstrate an influence of sex on UTI. This may be because of the insufficient sample size. Future studies that include a relatively large number of patients are required to further confirm the association between sex and UTI.

In summary, this study demonstrated that age and D-dimer are independent risk factors for UTI in ICH patients, and right-sided paralysis is a protective factor. The results indicate that attention should be paid to the effects of age, paralysis, and D-dimer on UTI for the treatment of patients. Based on the results, to better prevent or treat UTI effectively, we suggest the following: (1) sitting up rather than lying down in bed as much as possible; (2) drinking more water; (3) keeping the ureter open continuously, avoiding urine reflux, and removing the ureter as early as possible; (4) actively reducing D-dimer. These methods may help to prevent UTI. However, our results suggest that consciousness disorders, lower limb motor function, tracheotomies, pulmonary infection, indwelling catheters, leukocyte count, neutrophil proportion, serum sodium, uric acid, and FIB may also be related to UTI in patients after ICH. To provide more clinical evidence for effective prevention and intervention measures, the design of prospective studies with larger sample sizes that further analyze these potential influencing factors of UTI is urgent.

## Figures and Tables

**Table 1:** General data of the UTI group and the non-UTI group.

Group	*n*	Sex (*n*)		Age (year) (*x̄* ± SD)	Course (week) (*x̄* ± SD)	Paralysis side (*n*)			Location and type of bleeding (*n*)				Number of underlying diseases
		F	M			Left	Right	Both	A	B	C	D	
UTI	24	11	13	68.88 ± 9.157^a^	4.21 ± 1.744	9^b^	7^b^	8^b^	9	9	2	4	1.46 ± 0.779
Non-UTI	53	36	17	59.09 ± 6.775	5.19 ± 3.026	20	28	5	26	19	3	5	1.55 ± 0.889

F: female; M: male. Location and type of bleeding was defined as follows: left ICH, A type; right ICH, B type; ICH involved in the brainstem and ventricle, C type; subarachnoid hemorrhage (SAH), D type. For comparison with the non-UTI group, ^a^*P* < 0.01 and ^b^*P* < 0.05.

**Table 2:** Comparison of complications, secondary disorders, and rehabilitation interventions between the UTI group and the non-UTI group.

Group	Consciousness disorder		Tracheotomy		Retention catheterization		Pulmonary infection		Pressure sore		Venous thrombosis		Brunnstrom stage of lower limbs	Rehabilitation intervention	
	Yes	No	Yes	No	Yes	No	Yes	No	Yes	No	Yes	No		Yes	No
UTI	10^a^	14^a^	15^a^	9^a^	20^a^	4^a^	22^a^	2^a^	3	21	8	16	2.00 ± 1.103^b^	7	17
Non-UTI	7	46	7	46	9	44	24	29	1	52	10	43	2.66 ± 1.192	15	38

For comparison with the non-UTI group, ^a^*P* < 0.01 and ^b^*P* < 0.05.

**Table 3:** Comparison of blood indices between the two groups.

Group	Leukocyte count (*∗*10^9^)	Neutrophil proportion (%)	Platelet count (*∗*10^9^)	Albumin (g/L)	Globulin (g/L)	Blood sugar (mmol/L)	Creatinine (*μ*mol/L)	Uric acid (*μ*mol/L)	Sodium (mmol/L)	Potassium (mmol/L)	Calcium (mmol/L)	Magnesium (mmol/L)	D-dimer (mg/L)	Fibrinogen (g/L)
UTI	9.02 ± 3.524^b^	73.23 ± 9.982^a^	259.63 ± 95.399	34.41 ± 5.624	30.55 ± 4.887	5.91 ± 1.141	61.50 ± 22.437	221.55 ± 105.723^b^	136.54 ± 4.644^b^	3.99 ± 0.500	2.23 ± 0.130	0.85 ± 0.130	3.63 ± 4.723^a^	4.89 ± 1.667^a^
Non-UTI	7.19 ± 2.642	65.37 ± 12.692	241.47 ± 85.748	36.20 ± 4.869	29.00 ± 4.381	5.75 ± 1.835	67.77 ± 17.473	275.05 ± 96.745	139.51 ± 3.468	3.76 ± 0.489	2.25 ± 0.180	0.83 ± 0.086	1.52 ± 2.342	3.82 ± 1.180

For comparison with the non-UTI group, ^a^*P* < 0.01 and ^b^*P* < 0.05.

**Table 4:** Multivariate analysis of UTI in patients with cerebral hemorrhage.

Factors	*β*	Wald	*P*	OR	OR (95% CI)	
					Lower limit	Upper limit
Age	0.188	4.939	0.026	1.207	1.022	1.424
Paralysis side	—	4.914	0.086	—	—	—
Right: left	–3.917	4.851	0.028	0.020	0.001	0.650
Both: left	–0.622	0.172	0.678	0.537	0.028	10.131
Consciousness disorder (yes = 1, no = 0)	–1.890	1.199	0.274	0.151	0.005	4.450
Tracheotomy (yes = 1, no = 0)	2.700	2.472	0.116	14.874	0.514	430.543
Retention catheterization (yes = 1, no = 0)	1.803	2.304	0.129	6.067	0.591	62.249
Pulmonary infection (yes = 1, no = 0)	1.694	1.456	0.228	5.441	0.347	85.225
Brunnstrom stage of lower limbs	–0.669	1.290	0.256	0.512	0.162	1.625
Leukocyte count (*∗*10^9^)	0.416	2.010	0.156	1.516	0.853	2.694
Neutrophil proportion (%)	–0.096	1.110	0.292	0.909	0.760	1.086
Sodium (mmol/L)	0.026	0.033	0.856	1.026	0.776	1.358
Uric acid (*μ*mol/L)	0.006	0.796	0.372	1.006	0.993	1.018
D-dimer (mg/L)	0.338	3.918	0.048	1.403	1.003	1.961
Fibrinogen (g/L)	0.929	3.106	0.078	2.532	0.901	7.115

OR: odds ratio; CI: confidence interval; —: no data.

**Table 5:** Comparison of paralysis sides between the UTI group and the non-UTI group in female patients.

Group	*n*	Paralysis side (*n*)		
		Left	Right	Both
UTI	13	5	4	4
Non-UTI	17	7	7	3

*χ* ^2^ = 0.772; *P*=0.680.

**Table 6:** Comparison of paralysis sides between the UTI group and the non-UTI group in male patients.

Group	*n*	Paralysis side (*n*)		
		Left	Right	Both
UTI	11	4	3	4
Non-UTI	36	13	21	2

*χ* ^2^ = 6.873; *P*=0.032.

## Data Availability

All data generated or analyzed during this study are presented in the manuscript. Please contact the first author for access to the data presented in this study.
